# Role of CT imaging in addressing resectability issues in differentiated thyroid cancer: imaging-based Mahajan grading system for TI and ETE

**DOI:** 10.3389/fonc.2024.1382518

**Published:** 2024-09-13

**Authors:** Abhishek Mahajan, Shivam Rastogi, Shreya Shukla, Ujjwal Agarwal, Richa Vaish, Nivedita Chakrabarty, Renuka Ashtekar, Shonal Deokar, Atif Shaikh, Prathamesh Pai, Pankaj Chaturvedi, Sarbani Ghosh Laskar, Vasundhara Smriti, Swapnil U. Rane, Munita Bal, Asawari Patil, Neha Mittal, Vanita Noronha, Vijay Patil, Kumar Prabhash, Anil K. Dcruz

**Affiliations:** ^1^ Imaging Department, The Clatterbridge Cancer Centre NHS Foundation Trust, Liverpool, United Kingdom; ^2^ Faculty of Health and Life Sciences, University of Liverpool, Liverpool, United Kingdom; ^3^ Department of Radiodiagnosis and Imaging, Tata Memorial Hospital, Homi Bhabha National Institute, Mumbai, India; ^4^ Department of Radiodiagnosis and Imaging, MPMMCC and HBCH, Tata Memorial Hospital, Varanasi, India; ^5^ Department of Head and Neck Surgical Oncology, Tata Memorial Hospital, Homi Bhabha National Institute, Mumbai, India; ^6^ Department of Radiation Oncology, Tata Memorial Hospital, Homi Bhabha National Institute, Mumbai, India; ^7^ Department of Pathology, Tata Memorial Hospital, Homi Bhabha National Institute, Mumbai, India; ^8^ Department of Medical Oncology, Tata Memorial Hospital, Homi Bhabha National Institute, Mumbai, India

**Keywords:** thyroid cancer, differentiated papillary thyroid carcinoma, imaging, CT, US, diagnostic accuracy, extrathyroid extension, tracheal invasion

## Abstract

**Background and objective:**

Extrathyroidal extension (ETE) is the term used to describe the growth of the primary thyroid tumor beyond the thyroid capsule. ETE is a critical prognostic marker for thyroid tumors, necessitating accurate preoperative assessment. This study aims to evaluate the diagnostic performance of computed tomography (CT)-based grading for ETE and tracheal invasion (TI) for preoperative prediction in patients with differentiated papillary thyroid carcinoma (PTC) and compare the diagnostic accuracy with ultrasound (US).

**Materials and methods:**

This retrospective study was approved by our institutional review board. Preoperative US and CT were performed for 83 patients who underwent surgery for PTC between 1 January 2010 and 31 December 2020. The US and CT features of ETE and TI of each case were retrospectively and independently investigated by two radiologists. The diagnostic performances of US and CT, including their specificity, sensitivity, positive predictive value (PPV), and negative predictive value (NPV) for ETE, and their accuracy in predicting ETE and TI were analyzed. As per the grading for ETE on USG and CT, lesions were graded into three grades and Mahajan grading was also devised on CT to predict the TI and graded into four grades.

**Results:**

The accuracy and specificity of CT are relatively good for identifying tumor infiltration into the adjacent structures and range from 82% to 87% and 95% to 98%, respectively. It, however, has a low sensitivity, between 14.3% and 77.78%, when compared to US, which suggests that in case of any doubt regarding CT evidence of tumor infiltration into surrounding structures, additional clinical examination must be performed. CT showed better sensitivity (78%) and specificity (75%) in detecting TI compared to previous studies. The diagnostic accuracy of CT Mahajan grading was 91.5% with *p <*0.005 in the prediction of TI.

**Conclusion:**

Preoperative US should be regarded as a first-line imaging modality for predicting minimal ETE, and CT should be additionally performed for the evaluation of maximal ETE. The specificity and PPV of CT are higher than those of US in detecting overall ETE and TI of PTC. The US- and CT-based grading systems have the potential to optimize preoperative surgical planning.

## Introduction

Thyroid cancer is the most common endocrine malignancy, comprising ~2.1% of all cancer diagnoses worldwide. Differentiated thyroid cancers (DTCs) make up 90% of thyroid cancers, with papillary thyroid carcinoma (PTC) being the most common type, followed by follicular thyroid carcinoma (FTC) (1). Risk factors for advanced PTC include male sex, advanced age, larger tumors, and extrathyroidal extension (ETE) (2). Cross-sectional imaging plays a crucial role in presurgical staging, as ETE is a significantly unfavorable prognostic factor (3–6). ETE is the expansion of the primary thyroid tumor outside the thyroid capsule and may involve the trachea, larynx, jugular vein, carotid artery, esophagus, strap muscles, and recurrent laryngeal nerve. ETE can be classified as mild (histologically detected) or gross (preoperative or intraoperative evidence) (7–9). Previous descriptions of gross ETE characterized it as gross tumor invasion identified after surgery and verified by histopathologic analysis. Tumor invasion detected during pathologic examination that extended beyond the thyroid capsule was referred to as minor ETE. The absence of ETE indicates that neither intraoperative inspection nor histopathologic assessment detected any ETE. In cases where a large tumor invasion was suspected after surgery, but the tumor’s histopathologic evaluation revealed that it was limited to the thyroid and had not spread to the surrounding capsule, the condition was classified as simple adhesion. The updated eighth edition of the AJCC staging system has designated the T3b category for gross ETE involving the strap muscles alone, whereas gross ETE into the major neck structures is assigned the T4 category.

The most common techniques for locoregional assessment are ultrasound (US), computed tomography (CT), and magnetic resonance imaging (MRI) (10). When ETE is suspected on the basis of symptoms or physical examination, cross-sectional imaging using CT or MRI is crucial as ETE is linked to a higher incidence of distant metastases (11, 12). CT with contrast provides a detailed evaluation of the thyroid tumor’s relationship to neck tissues and is ideal for surgical planning and assessing lymph node involvement (13).

Tracheal invasion (TI) is common among individuals with tumor invasion into major neck structures, occurring in 35%–60% of cases, followed by esophageal and laryngeal invasion. Approximately 7%–10% of thyroid carcinoma cases involve laryngotracheal invasion. TI stages from papillary carcinoma are stage 1 (limited to glandular parenchyma), stage 2 (invades the tracheal cartilage or intercartilaginous tissue), stage 3 (invades the tracheal mucosa’s lamina propria without penetrating), and stage 4 (fully invades the trachea with visible ulcerations or neoplastic vegetations).

ETE is a strong marker influencing surgical margins and the need for adjuvant therapy. In well-differentiated thyroid cancer, the incidence of ETE varies from 5% to 34% (14). In the case of minor ETE, near-total tumor excision with adjuvant treatment offers survival rates comparable to extensive resection. However, extensive local resection increases the survival rate and decreases local recurrence when ETE is present (15).

Although CT is the preferred imaging modality for evaluating thyroid tumors with TI, there is a dearth of research on CT-based grading of TI. This retrospective study aims to evaluate the diagnostic performance of CT-based grading for ETE and TI for preoperative prediction in patients with differentiated PTC and to evaluate the diagnostic performance of CT-based grading systems.

## Materials and methods

### Materials

**Figure 1:**
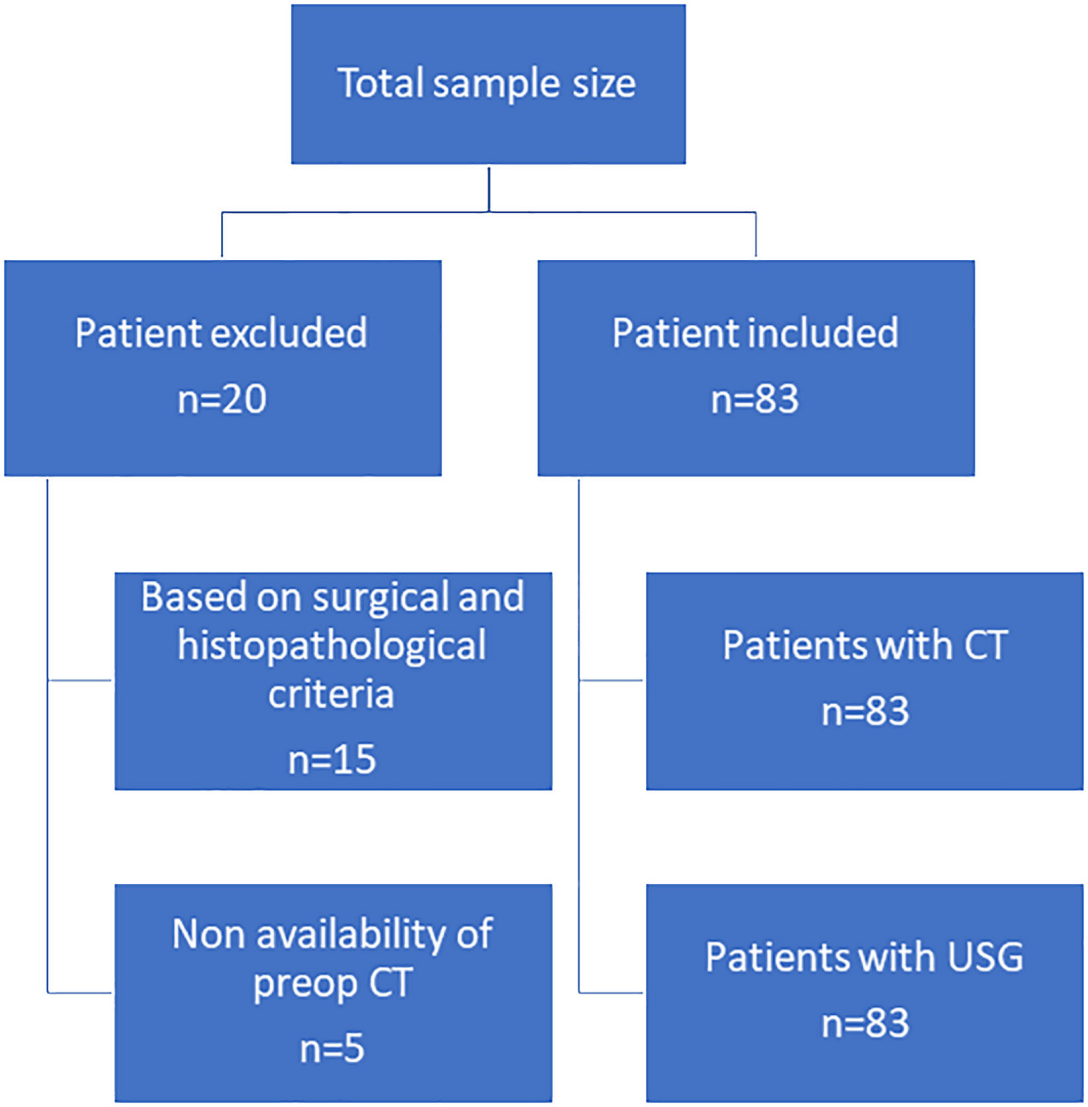
Flowchart showing the distribution of cases.

This retrospective study was conducted with the approval of the institutional review board and a waiver of patient informed consent. We retrospectively reviewed cases of 103 patients with differentiated PTC. Twenty of these patients were excluded on the basis of the following: the extent of disease was not fully explored on the basis of surgical and histopathologic findings due to unavailability of surgical details or detailed histopathological report (15 patients) and preoperative CT examinations were not performed at our center (5 patients). Thus, our study sample consisted of 83 differentiated papillary thyroid tumors of the thyroid [one tumor in 59 patients and two tumors (one in each lobe) in 12 patients]. All patients underwent complete or near-complete resection of the primary tumor; 62 of these patients had ETE according to the histopathological findings (**Figure 1**).

### Image analysis

All patients underwent preoperative imaging by USG and CT. All the USG and CT images were retrospectively interpreted and individually reviewed. All images were presented in an anonymous random manner on a picture archiving and communication system (PACS) workstation. The US images available on PACS were interpreted for ETE, which was categorized based on contact with the tumor and disruption of the thyroid capsule. When a patient had several thyroid masses, radiologists were informed about the location of the thyroid cancer but were otherwise blinded to the initial CT reports and surgical and pathologic findings. This action was primarily done to shorten the review period for the evaluation of thyroid cancer’s extrathyroidal invasion. The mean values of the two reviewers were used for the continuous data, and the final interpretation for the invasion grade was based on the radiologists’ consensus. Whenever there was a discrepancy in data interpretation in a particular category between the two radiologists, a third radiologist reviewed the cases, and the opinion of the majority was considered as the final decision. Interobserver agreement was moderate to good with a mean kappa value of 0.76 (range, 0.65–0.88).

**Figure 2:**
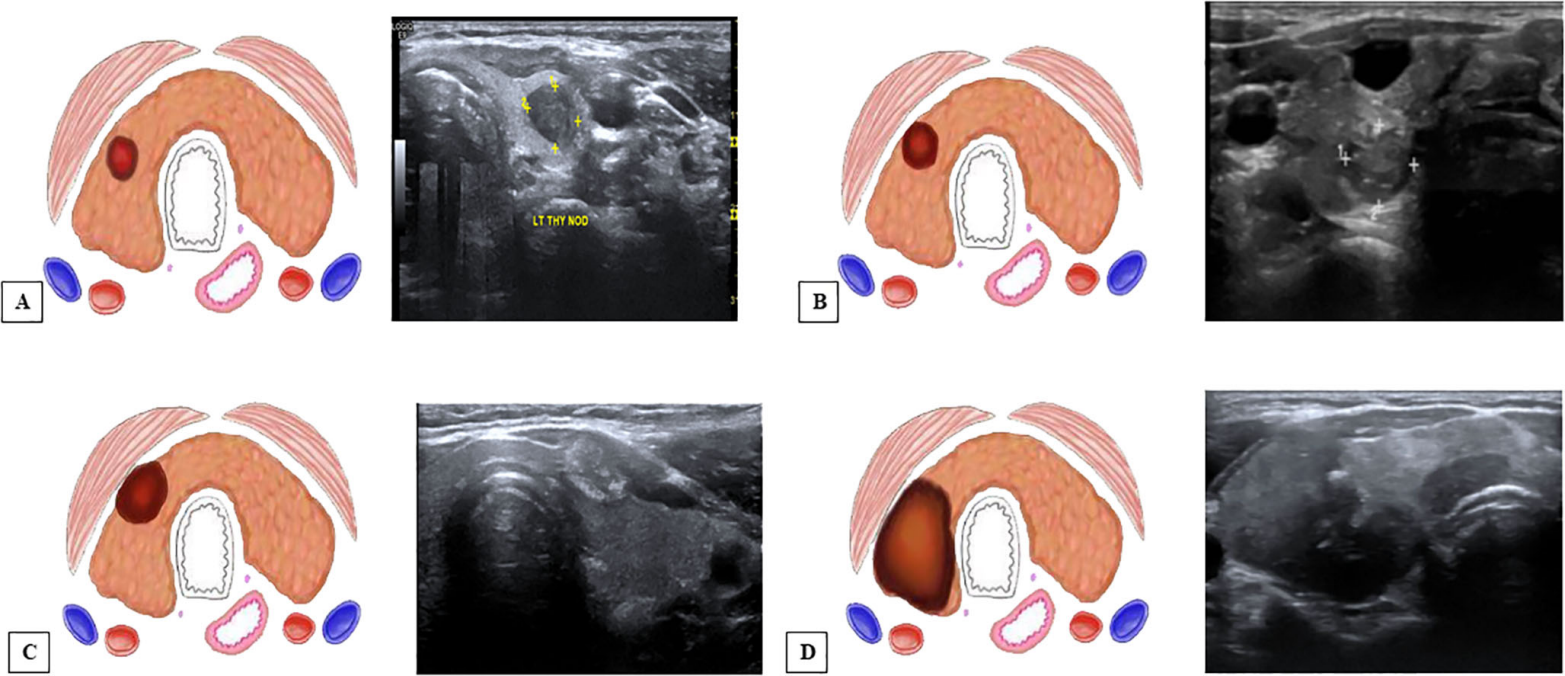
Grading system for ultrasound (US) and computed tomography (CT) for extrathyroidal extension (ETE) (12). **(A)** US and an illustration showing ETE grade 0—tumor which was completely enveloped by thyroid parenchyma. **(B)** ETE grade 1—tumor in which the percentage of the tumor perimeter in contact with the thyroid capsule was 1%–25%. **(C)** ETE grade 2—tumor in which contact with the capsule was 25%–50%. **(D)** ETE grade 3—tumor in which contact with the capsule was >50%.

**Figure 3:**
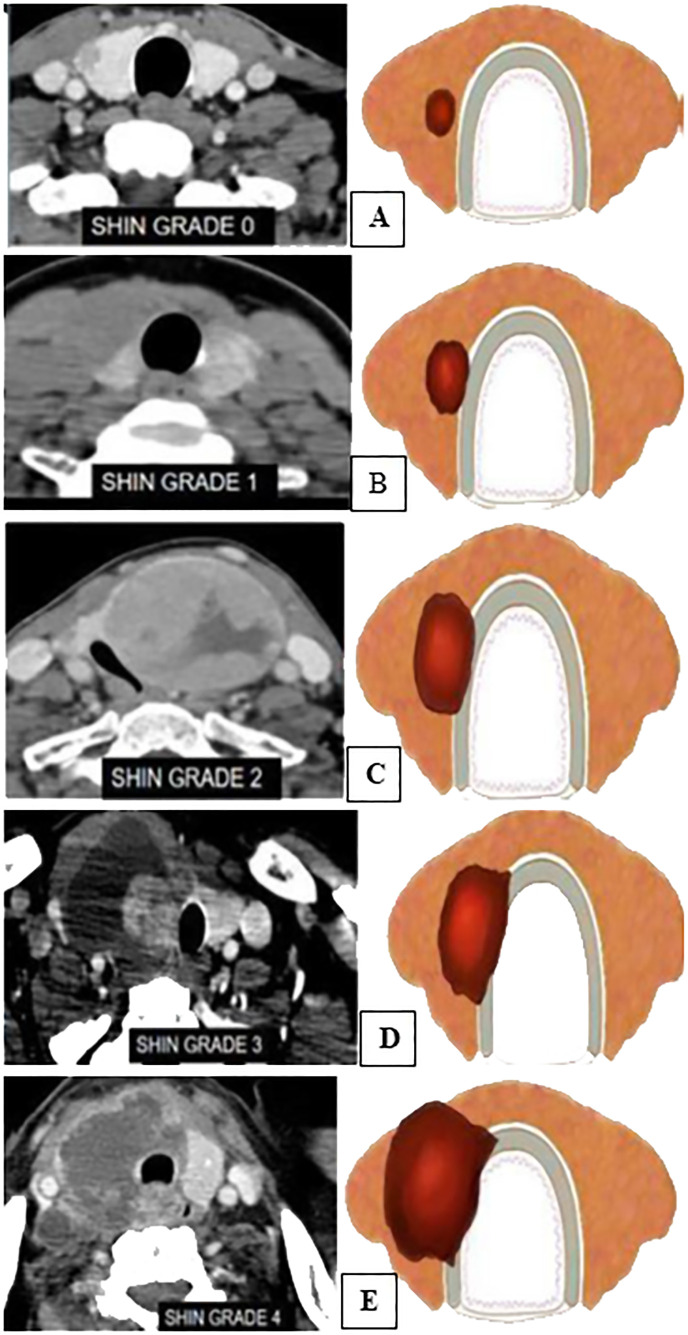
Mahajan grading for tracheal invasion (12). **(A)** CECT and an illustration showing grade 0→5 mm distance between the tumor and the trachea. **(B)** Grade 1—disease abuts the external perichondrium. **(C)** Grade 2—disease invades into the cartilage +/− destruction. **(D)** Grade 3—disease extends into the tracheal mucosa with no elevation/penetration of the mucosa. **(E)** Grade 4—disease is full-thickness invasion with expansion of the tracheal mucosa with a bulge.

**Figure 4:**
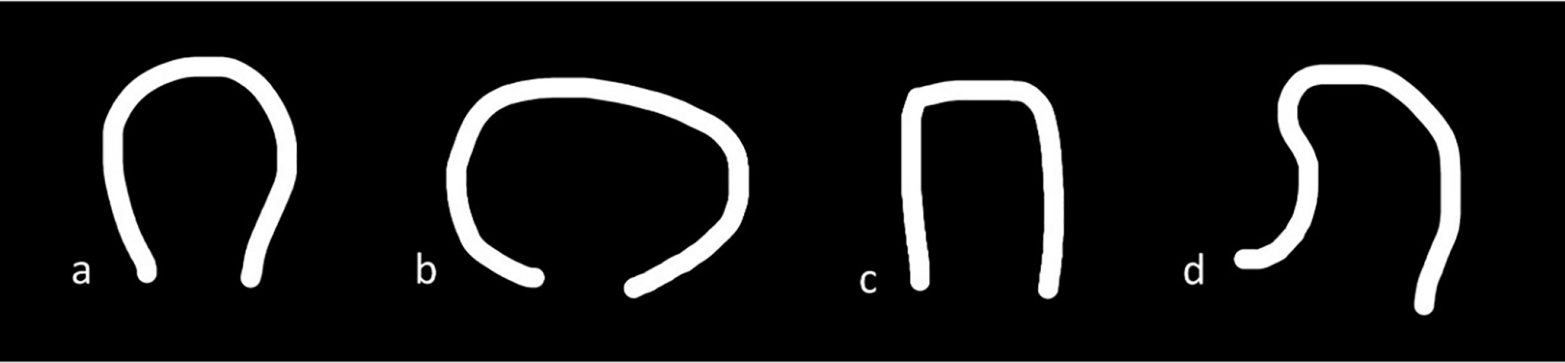
Tracheal deformity shapes. **(A)** Grade 0—horseshoe. **(B)** Grade 1—elliptical or circular configuration. **(C)** Grade 2—trachea with a locally straightened wall. **(D)** Grade 3—trachea with an inward concave deformity.

**Figure 5:**
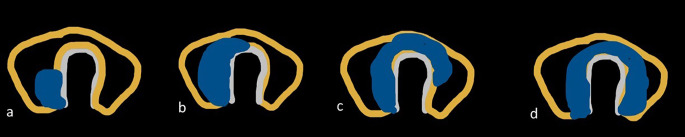
Degree of encirclement of the tumor circumference with that of the trachea. **(A)** Grade I, 0°–89°. **(B)** Grade II, 90°–179°. **(C)** Grade III, 180°–269°. **(D)** Grade IV, 270°–360°.

The CT findings were independently evaluated with regard to the characteristics of the tumor, i.e., size, enhancement, cystic change, calcification, and mediastinal extension. The other parameters evaluated were as follows:

1. Grading for extrathyroid extension: grade 0 ETE, a tumor which was completely enveloped by thyroid parenchyma; grade I ETE, a tumor in which the percentage of the tumor perimeter in contact with the thyroid capsule was 1%–25%; grade II, a tumor in which the contact with the capsule was 25%–50%; and grade III, a tumor in which the contact with the capsule was >50% (**Figure 2**).
2. Grading was devised based on CT to predict the TI: grade 0, >5 mm distance between the tumor and the trachea; grade I, disease abuts external perichondrium; grade II, disease invades into the cartilage +/− destruction; grade III, disease extends into the tracheal mucosa with no elevation/penetration of the mucosa; and grade IV, disease shows full-thickness invasion with expansion of the tracheal mucosa with a bulge (**Figure 3**).
3. Tracheal deformity shapes were classified as follows: grade 0, horseshoe; grade 1, elliptical or circular configuration; grade 2, trachea with a locally straightened wall; and grade 3, trachea with an inward concave deformity. When the trachea was slightly longer in the anterior–posterior dimension than in the transverse dimension, it was referred to as having a horseshoe shape. When the trachea’s transverse and anteroposterior diameters were almost identical, the elliptical or circular shape was determined. When the trachea was locally flattened along the anterior curve, it was defined to have a “locally straightened shape” (**Figure 4**).
4. The degree of encirclement of the tumor circumference with that of the trachea with loss of intervening fat plane was classified into four consecutive grades: grade I, 0°–89°; grade II, 90°–179°; grade III, 180°–269°; and grade IV, 270°–360° (**Figure 5**).

These above parameters were correlated with their gold standard histopathological reports. Imaging analysis characterized the structures involved as either present or absent. The ETE on histopathology was classified as minimal (or minor) if the tumor extended beyond the thyroid capsule and maximal (or gross) if it involved the surrounding structures.

A new risk scoring system using Mahajan grading was devised based on CT findings for prediction of TI (**Tables 1**, **2**). A minimum score of 0 and a maximum score of 8 were given. The CT parameters used were Mahajan grading for TI, tracheal deformity, degree of encirclement, and tracheo-esophageal groove involvement. The US parameters used were the size of the thyroid lesion, angle of contact between the tumor and the trachea, grade of ETE, contour bulge, capsular disruption, and replacement of strap muscle by the tumor.

**Table 1:** Computed tomography (CT)-based grading system.

Imaging Characteristic	0	1	2	3	4
Tracheal deformity	Grade 1	≥Grade 2			
Degree of encirclement	≤Grade 2	≥Grade 3			
Degree of invasion	Grade 0	≥Grade 1			
TE groove involvement	Absent	Present			

**Table 2:** Ultrasound (US)-based grading system.

Imaging Characteristic	0	1	2	3
Max. tumor diameter	<2 cm	≥2 cm		
Angle of contact	Acute angle	Obtuse angle		
Contour bulge	Absent	Present		
Capsular disruption	Absent	Present		
Grade of ETE	Grade 0	Grade 1	Grade 2	Grade 3
Strap muscle involvement	Absent	Present		

### Statistical analysis

All statistical analyses were performed using Statistical Package for Social Sciences (SPSS) software, version 21.0. The statistical significance of the relationship between USG and CT findings—such as contact and disruption of the capsule with ETE—was assessed using chi-square testing. The statistical significance of variations in the mean values of continuous variables was assessed using the Student’s *t*-test. An examination of the receiver-operating characteristic (ROC) curve was conducted to assess the accuracy with which US and CT findings predicted ETE. Statistical significance was defined as a value <0.05. To perform statistical analysis, 2 × 2 tables were created for CT diagnoses of extrathyroidal invasion to surrounding structures that were true positive, false positive, true negative, and false negative. Following surgery, the pathological results were compared with the findings from US and CT scans, and it was determined that the pathological findings and the ETE found by US and CT scans corresponded. Based on the findings, the diagnostic accuracy rates of the modalities were computed, including the sensitivity, specificity, positive predictive value (PPV), and negative predictive value (NPV). The independent *t*-test and the *χ* ^2^ test were used to compare continuous and categorical variables in terms of clinical features. *p*-values less than 0.05 were deemed statistically significant. A review and correlation between CT imaging results and surgical and pathologic findings were conducted on 83 patients diagnosed with thyroid cancer. The most relevant CT imaging markers for predicting TI were identified using a logistic regression model. In order to predict ETE, a grading system was developed based on CT and USG findings. For categorical variables, frequency and percentage were used in a descriptive analysis of the data. Utilizing the chi-square test, the relationship between the two variables was examined. ROC curves and area under the curve (AUC) were utilized to display the accuracy of several representative models. The Youden index approach was used to generate the ROC cutoffs. A value of *p <*0.05 was deemed statistically significant for all two-sided statistics.

## Results

### Patient characteristics

The demographic characteristics of the patients in our study are summarized in **Table 3**. The average age of presentation of papillary thyroid cancer was 49 years with a range of 19 to 88 years. Majority of the patients in our study were between 30 and 39 years old. Among the 83 patients, a total of 48 women and 35 men had *p*-values of 0.342, suggesting that there is no positive correlation between the presence of ETE and gender.

**Table 3:** Demographic and clinicoradiopathologic characteristics of patients with differentiated papillary thyroid cancer.

Factors	Number (*N*)	Percentage (%)
Gender		
Male	35	42.2
Female	48	57.8
Location of tumor		
Right lobe	35	42.2
Left lobe	21	25.3
Isthmus	3	3.6
Bilateral lobe	24	28.9
**Age (mean)**	48.9 ± 1.72 years	
Clinical symptoms		
Neck swelling	82	98.0
Hoarseness of voice	2	2.4
Respiratory distress	3	3.6
Hemoptysis	0	0
CT features		
Mean size (AP × TR × CC)	3.49 ± 1.7 × 2.99 ± 1.5 × 3.97 ± 2.15	
Multifocality		
Unifocal	64	77
Multifocal	19	23
Enhancement		
Heterogeneous	68	82
Homogeneous	15	18
Cystic change	55	66
Calcifications	47	56
ETE on CT	75	90
Mediastinal extension	23	28
Strap muscle	35	42
TE groove	33	40
Metastatic nodal involvement	55	66
Surgery		
Hemithyroidectomy	3	3.6
Total thyroidectomy	80	96.4
Pathological findings		
ETE on HPR	62	74.7
pStrap muscle	34	41.0
pTrachea	14	17
pEsophagus	5	6
pRLN	16	19
Metastatic node	58	70

### Imaging characteristics

#### USG-based findings

The frequency of USG-based imaging findings in relation to ETE on histopathology is summarized in **Table 4**. Odds ratios of several US findings were calculated. The diagnostic characteristics, including sensitivity, specificity, accuracy, PPV, and NPV, were calculated according to the sonographic criteria for predicting ETE as shown in **Table 5**. Values of 95% and 92% for specificity and PPV, respectively, were the highest in the US finding of grade 2, where there is more than 25% contact with the adjacent capsule. The diagnostic accuracy value was the highest with grade 3, where there is more than 50% contact with the adjacent capsule. The *p*-value of the contour bulge and capsular disruption on US with ETE on pathological investigations was <0.05, showing a significant correlation between them. The PPV values were the highest with ETE and US feature of strap muscle replacement. ETE on HPR was present in 37 out of 42 patients in whom the thyroid cancers had formed an obtuse angle with the trachea (sensitivity, 59.68%; specificity, 76.19%; PPV, 88.1%; NPV, 39.02%; diagnostic accuracy, 64%). Thus, there was a significant correlation between the angle of contact and ETE with *p <*0.05.

**Table 4:** Frequency of ultrasound (US)-based imaging findings in relation to extrathyroid extension on histopathology.

Sonographic findings	ETE on HPR present 74.7% (*n* = 62)	ETE on HPR absent 25.3% (*n* = 21)	*p*-value
Extrathyroid extension			
Grade 0: 7.2% (*n* = 6)	1.6% (*n* = 1)	23.8% (*n* = 5)	0.000687
Grade 1: 7.2% (*n* = 6)	1.6% (*n* = 1)	23.8% (*n* = 5)	0.000687
Grade 2: 15.7% (*n* = 13)	19.4% (*n* = 12)	4.8% (*n* = 1)	0.111778
Grade 3: 69.9% (*n* = 58)	77.4% (*n* = 48)	47.6% (*n* = 10)	0.005332
Contour bulge			
Present: 75.9% (*n* = 63)	83.9% (*n* = 52)	52.4% (*n* = 11)	0.0035
Absent: 24.1% (*n* = 20)	16.1% (*n* = 10)	47.6% (*n* = 10)	
Capsular disruption			
Present: 67.5% (*n* = 56)	77.4% (*n* = 48)	38.1% (*n* = 8)	0.0008
Absent: 32.5% (*n* = 27)	22.6% (*n* = 14)	61.9% (*n* = 13)	
Replacement of strap muscle			
Present: 45.8% (*n* = 38)	51.6% (*n* = 32)	28.6% (*n* = 6)	0.066
Absent: 54.2% (*n* = 45)	48.4% (*n* = 30)	71.4% (*n* = 15)	
Angle of contact			
Acute: 49.4% (*n* = 41)	40.3% (*n* = 25)	76.2% (*n* = 16)	0.0044
Obtuse: 50.6% (*n* = 42)	59.7% (*n* = 37)	23.8% (*n* = 5)	

**Table 5:** Diagnostic accuracy of ultrasound (US)-based imaging findings in relation to extrathyroid extension on histopathology.

Sonographic findings	Sensitivity (%)	Specificity (%)	PPV (%)	NPV (%)	Accuracy (95% CI) (%)
Grade 0	23.81%	98.39%	83.33%	79.22%	79.52%
Grade 1	23.81%	98.39%	83.33%	79.22%	79.52%
Grade 2	19.35%	95.24%	92.31%	28.57%	38.55%
Grade 3	77.42%	52.38%	82.76%	44%	71.08%
Contour bulge	83.87%	47.62%	82.54%	50%	74.7%
Capsular disruption	77.42%	61.9%	85.71%	48.15%	73.49%
Replacement of strap muscle	51.61%	71.43%	84.21%	33.33%	56.63%
Angle of contact	59.68%	76.19%	88.1%	39.02%	63.86%

#### CT-based findings

The CT findings of 83 patients were interpreted and data were collected in a synoptic format which included characteristics of the thyroid lesion like enhancement, cystic changes, calcification, mediastinal extension, grades of ETE, TE groove involvement, and Mahajan grading. The frequency of CT-based imaging findings in relation to ETE on histopathology is summarized in **Table 6**.

**Table 6:** Frequency of computed tomography (CT)-based imaging findings in relation to extrathyroid extension on histopathology.

CT findings	ETE on HPR present 74.4% (*n* = 62)	ETE on HPR absent 25.3% (*n* = 21)	*p*-value
Enhancement			
Homogeneous: 18.1% (*n* = 15)	17.7% (*n* = 11)	19.0% (*n* = 4)	0.893
Heterogeneous: 81.9% (*n* = 68)	82.3% (*n* = 51)	81.0% (*n* = 17)	
Cystic changes			
Present: 66.3% (*n* = 55)	67.7% (*n* = 42)	61.9% (*n* = 13)	0.624
Absent: 33.7% (*n* = 28)	32.3% (*n* = 20)	38.1% (*n* = 8)	
Calcification			
Present: 56.6% (*n* = 47)	62.9% (*n* = 39)	38.1% (*n* = 8)	0.084
Absent: 43.4% (*n* = 36)	37.1% (*n* = 23)	61.9% (*n* = 13)	
Mediastinal extension			
Present: 27.7% (*n* = 23)	29.0% (*n* = 18)	23.8% (*n* = 5)	0.643
Absent: 72.3% (*n* = 60)	71.0% (*n* = 44)	76.2% (*n* = 5)	
ETE on CT			
Grade 0: 9.6% (*n* = 8)	1.6% (*n* = 1)	33.3% (*n* = 7)	0.000
Grade I: 6.0% (*n* = 5)	3.2% (*n* = 2)	14.3% (*n* = 3)	0.065
Grade II: 18.1% (*n* = 15)	22.6% (*n* = 14)	4.8% (*n* = 1)	0.066
Grade III: 66.3% (*n* = 55)	72.6% (*n* = 45)	47.6% (*n* = 10)	0.036
TE groove involvement			
Present: 39.8% (*n* = 33)	50.0% (*n* = 31)	9.5% (*n* = 2)	0.001
Absent: 60.2% (*n* = 50)	50% (*n* = 31)	90.5% (*n* = 19)	
Mahajan grading for TI			
Grade 0: 22.9% (*n* = 19)	19.4% (*n* = 12)	33.3% (*n* = 7)	0.187
Grade I: 44.6% (*n* = 37)	41.9% (*n* = 26)	52.4% (*n* = 11)	0.405
Grade II: 8.4% (*n* = 7)	8.1% (*n* = 5)	9.5% (*n* = 2)	0.097
Grade III: 15.7% (*n* = 13)	19.4% (*n* = 12)	4.8% (*n* = 1)	0.111
Grade IV: 8.4% (*n* = 7)	11.3% (*n* = 7)	0% (*n* = 0)	0.248

The diagnostic performance of the CT findings including sensitivity, specificity, accuracy, PPV, NPV, and diagnostic accuracy for predicting absent, minimal, and maximal ETE based on the pathosurgical ETE is summarized in **Table 7**. There was a positive correlation between patients with involvement of the TE groove and maximal ETE. In almost all the patients where maximal ETE was present on the surgical pathology report, the TE groove was involved in CT. Patients graded into grades II, III, and IV were found to have maximal ETE as shown in **Table 6**.

**Table 7:** Diagnostic performance of computed tomography (CT) in assessing the extrathyroidal extension (ETE) of papillary thyroid carcinoma (PTC) based on pathosurgical classification.

ETE	Sensitivity (%)	Specificity (%)	PPV (%)	NPV (%)	Accuracy (%)	*p*-value
Absent ETE	24.19%	28.57%	50%	11.32%	25.3%	0.00009855
Minimal ETE	9.68%	95.24%	85.71%	26.32%	31.33%	0.048702
Maximal ETE	66.13%	76.19%	89.13%	43.24%	68.67%	0.000746

Overall, the involvement of the TE groove and grades of ETE on CT showed a significant correlation with the TI with *p*-values <0.05. Grading was proposed to correlate with the diagnostic performance of CT-based invasion of the trachea by the thyroid cancers. Grade IV had the highest specificity, sensitivity, PPV, NPV, and accuracy values of 50%, 100%, 100%, 90.1%, and 91.5%, respectively. Patients having TI showed a significantly higher incidence of grade IV (*p* < 0.001) in comparison to those with the other grades. There was a gradual increase in diagnostic accuracy from grade 0 to grade IV. The sensitivity and specificity of grade III tracheal deformity (100%; 100%), grade III degree of encirclement (90.5%; 91.6%), and grade III invasion (90.5%; 94%) were significant with *p <*0.05, when correlated with the ETE on HPR, as shown in **Table 8**.

**Table 8:** Diagnostic performance of computed tomography (CT) findings in assessing the tracheal invasion in papillary thyroid carcinoma (PTC).

CT findings	Sensitivity (%)	Specificity (%)	PPV (%)	NPV (%)	*p*-value	accuracy (95% CI) (%)
Tracheal deformity						
Grade I	78.26%	92.86%	98.18%	46.43%	<0.00001	80.72%
Grade II	50%	79.71%	33.33%	88.71%	0.0197	74.7%
Grade III	42.86%	98.55%	85.71%	89.47%	0.0197	89.47%
Degree of encirclement						
Grade 0	27.54%	100%	100%	21.87%	0.323	39.76%
Grade I	7.25%	73.91%	100%	17.95%	0.583	22.89%
Grade II	14.29%	73.91%	10%	80.95%	0.943	63.86%
Grade III	42.86%	73.91%	25%	86.44%	0.206	68.67%
Grade IV	42.86%	86.96%	40%	88.24%	0.672	79.52%
Degree of invasion						
Grade 0	69.57%	100%	100%	40%	<0.001	74.7%
Grade I	8.7%	78.57%	66.67%	14.86%	0.162	20.48%
Grade II	28.57%	78.26%	21.05%	84.37%	0.579	69.88%
Grade III	50%	100%	100%	90.79%	0.000	91.57%

A logistic regression model revealed that involvement of the trachea (*p* < 0.001) and involvement of the esophagus in histopathological studies (*p* < 0.05) were significant factors for predicting the involvement of TE groove on CT. The involvement of the recurrent laryngeal nerve was not significant in predicting the involvement of the TE groove. Of the three factors, the involvement of the trachea and recurrent laryngeal nerve was more accurate than the involvement of the esophagus. The pathological involvement of the esophagus had a high NPV of 98% when involvement was given on CT. Using the three predictors, the combined criteria of the involvement of the trachea, esophagus, and recurrent laryngeal nerve on histopathology produced 71% accuracy and 82% specificity as shown in **Table 9**.

**Table 9:** Diagnostic performance of computed tomography (CT) in assessing the tracheo-esophageal groove involvement in papillary thyroid carcinoma (PTC).

CT findings	Sensitivity	Specificity	PPV	NPV	*p*-value	Accuracy
pTrachea	78.57%	75.82%	33.33%	95.83%	0.00113	76.19%
pEsophagus	80%	62.82%	12.12%	98%	0.057878	63.86%
pRecurrent laryngeal nerve	87.5%	64.18%	67.12%	86%	0.133567	74.8%
Combination of all the three above	54.55%	82%	66.67%	73.21%	0.000	71.08%

A logistic regression model showed that the only significant features predicting TI were soft tissue in the cartilage (*p* < 0.001), intraluminal mass (*p* < 0.001), and the circumference of 180° or more (*p* = 0.001) of the tumor surrounding the trachea. Out of these three factors, accuracy was the highest (90% accuracy) for soft tissue in the cartilage with a sensitivity of 77% and a specificity of 100%. The sensitivity of CT for diagnosing intraluminal mass was low but without any false-positive diagnoses (100% specificity). The addition of Mahajan grade III and grade IV with a circumference of 180° or more of the tumor surrounding the trachea yielded the highest accuracy (74%) with a sensitivity and specificity of 100% and 70%, respectively.

Strap muscle involvement on HPR was present in 32 out of 34 patients in whom thyroid cancers had infiltrated the strap muscles or there was loss of fat planes with the strap muscles (sensitivity, 94.12%; specificity, 93.88%; PPV, 91.43%; NPV, 95.83%; diagnostic accuracy, 93.98%). Thus, there was a significant correlation between the strap muscle involvement on histopathology and its involvement on CT with a *p*-value <0.05. There was a significant correlation between the presence and absence of ETE and the surgical approach (hemithyroidectomy versus total thyroidectomy) with a *p*-value of 0.019.

## Discussion

The overall prognosis and survival of well-differentiated PTC is excellent. The incidence of invasion of the esophagus and laryngotracheal structures by well-differentiated thyroid carcinoma ranges between 1% and 16% even though the thyroid is in close vicinity of these upper aerodigestive tract (ADT) structures (16, 17). The risk of morbidity and mortality can rise with infiltration of the ADT. Well-encapsulated tumors have a 10-year overall survival rate of 91%, which drops to 45% in those having ETE, which is known to be a poor prognostic indicator (18). Thyroid aberrations are commonly initially detected on other cross-sectional modalities such as CT or MRI even though US is the first-line imaging modality for a palpable thyroid nodule or in those with a known thyroid malignancy (19). In our study, most of the patients were women with a mean age at diagnosis of 48.9 years, presenting with complaints of neck swelling. In our study, the specificity, PPV, and NPV for the prediction of ETE were higher in grades 0, I, and II when compared to a study by Kwak et al. The specificity of grade III in our study was 53%, whereas it was 92.4% in the latter. The sensitivity of grade III ETE was higher than that in the study by Kwak et al. (20).

According to the study by Hu et al., the clinicopathological features of patients with minimal and maximal ETE were based on the pathosurgical classification of ETE (21). A significant correlation was found between the mean age, size of the primary tumor, and surgical approach of the patients with minimal and maximal ETE with a *p*-value of less than 0.05. However, no statistical difference was seen in the clinicopathological characteristics like sex, lymph nodal metastasis, and tumor location of PTCs with minimal and maximal ETE.

In our series, statistically significant features predicting TI on CT were soft tissue in the cartilage, intraluminal mass, and the degree of encirclement of tracheal circumference by the tumor. Our study, in concordance with other documented studies in the literature, showed a high specificity (100% specificity) of TI in the presence of intraluminal mass, and this finding always indicated pathological deep tumor infiltration, either in the submucosal areas or in the mucous membrane. Similarly, the study by Wang et al. showed comparable results with a high specificity (100% specificity) of TI in the presence of intraluminal mass and a high accuracy of 87% for TI in the presence of soft tissue signal in the cartilage (22).

**Figure 6:**
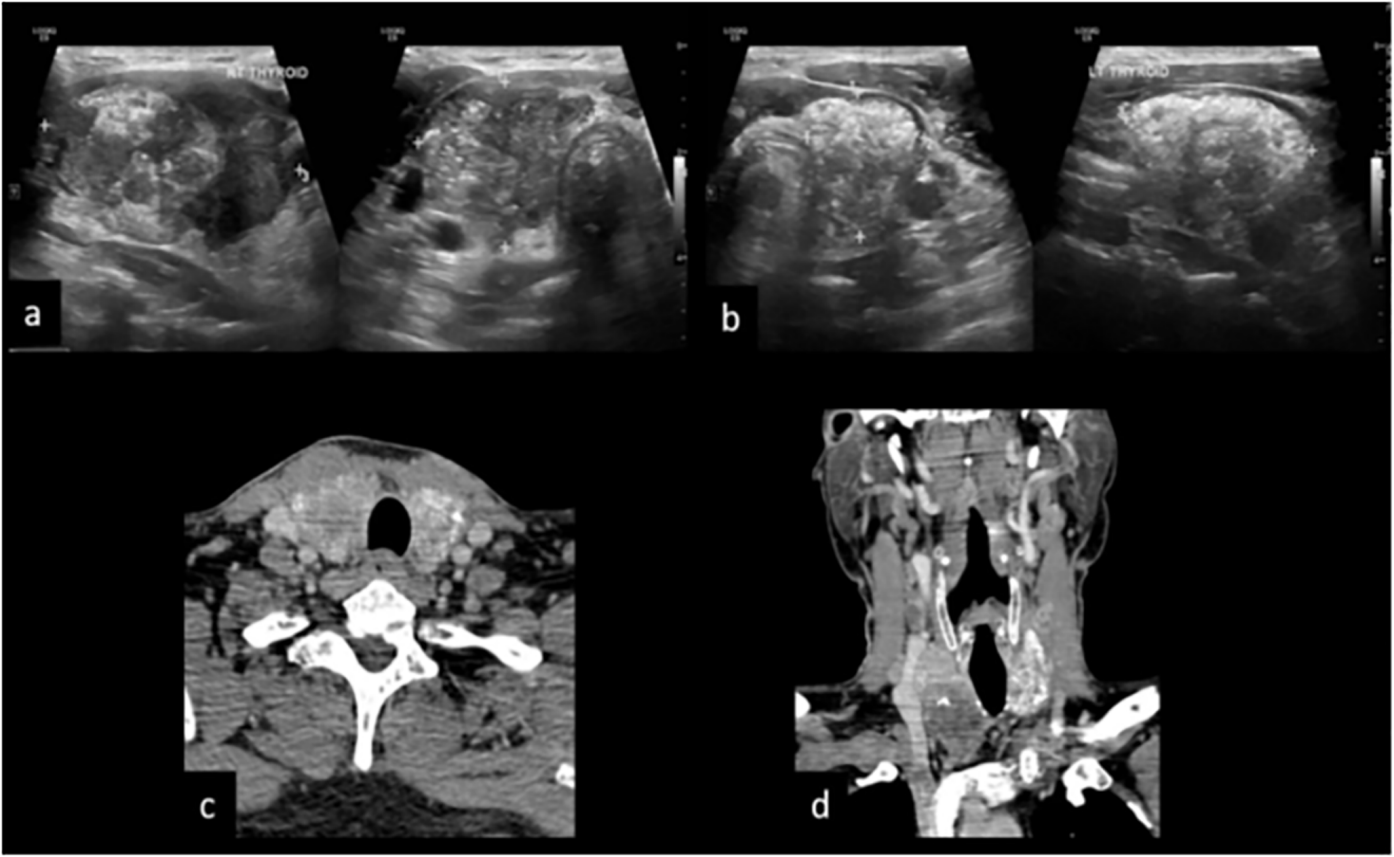
A 50-year male patient presented with anterior neck swelling since 4 months. **(A, B)** US reveals diffusely enlarged thyroid gland replaced completely by ill-defined solid hypoechoic mass with microcalcifications and macrocalcifications, both central and peripheral vascularity and TIRADS 5. **(C)** CT axial image shows heterogeneously enhancing mass replacing both the lobes of the thyroid gland with calcifications and cystic areas grade III, tracheal invasion grade I, and degree of encirclement grade IV with TE groove involvement. **(D)** CT coronal image shows mediastinal extension of the mass and metastatic cervical adenopathy.

CT showed a sensitivity of 78% and a specificity of 75% for detecting TI, which was better compared to the previous studies by Seo et al. and H. Kim et al. (23, 24). Detection of the early stage of TI is difficult on cross-section as tracheal adventitia is thin and there is degradation of image quality due to motion artifact, particularly when the PTC is small. Our study showed higher sensitivity (80%) of CT for evaluation of esophageal invasion. Our study had lower sensitivity and specificity in the evaluation of involvement of the recurrent laryngeal nerve. **Figure 6** shows an illustration of the case showing the application of grading.

Discussion of CT-based TI grading is lacking in the current literature. We tried to cumulate the factors which can predict TI which would guide surgical management. We propose a new Mahajan grading system with the inclusion of the parameters of the old grading system for risk stratification of patients with TI. The limitations of our study include 1) selection bias due to the retrospective nature of the study as the included patients underwent preoperative CT on clinical suspicion of ETE. 2) The sample size of the tumors infiltrating the trachea, esophagus, major vessels, and RLN was small, and this low prevalence could have limited the calculated descriptive statistics for the involvement of each of the structures by ETE. 3) Reliance on a retrospective design did not allow for the direct comparison of individual grades on imaging with the pathological Shin grades. A prospective study with increased axial slicing of the tumor would be required to achieve this comparison. 4) Lastly, long-term survival, recurrence rate, and its implication on adjuvant therapy were not evaluated.

## Conclusion

CT is a valuable imaging modality for the assessment of ETE although its effectiveness is limited by reduced sensitivity. Preoperative US should be regarded as a first-line imaging modality for predicting minimal ETE, and CT should be additionally performed for the evaluation of maximal ETE in cases with large tumors which are incompletely imaged on US, sonographic suspicion for ETE, or tumors showing direct contact with the capsule. The specificity and PPV of CT are higher than US in detecting overall ETE for PTC. Application of the Mahajan grading and scoring systems for prognostic high-risk groups results in a better selection of initial treatment and postoperative follow-up and, hence, is of high value.

## Data Availability

The original contributions presented in the study are included in the article/supplementary material. Further inquiries can be directed to the corresponding author.

## References

[1] Mahajan A Vaish R Arya S Sable N Pande S Paul P. Diagnostic performance of thyroid multimodal-imaging comprehensive risk stratification scoring (TMC-RSS) system in characterising thyroid nodules. J Clin Oncol. (2017) 35:e17588–8. doi: 10.1200/JCO.2017.35.15_suppl.e17588

[2] Hu S Zhang H Sun Z Ge Y Li J Yu C. Preoperative assessment of extrathyroidal extension of papillary thyroid carcinomas by ultrasound and magnetic resonance imaging: a comparative study. Radiol Med (Torino). (2020) 125:870–6. doi: 10.1007/s11547-020-01184-0 32249390

[3] Cushing SL Palme CE Audet N Eski S Walfish PG Freeman JL. Prognostic factors in well-differentiated thyroid carcinoma. Laryngoscope. (2004) 114:2110–5. doi: 10.1097/01.mlg.0000149442.22393.e2 15564829

[4] Jukkola A Bloigu R Ebeling T Salmela P Blanco G. Prognostic factors in differentiated thyroid carcinomas and their implications for current staging classifications. Endocr Relat Cancer. (2004) 11:571–9. doi: 10.1677/erc.1.00826 15369456

[5] McCaffrey TV Bergstralh EJ Hay ID. Locally invasive papillary thyroid carcinoma: 1940-1990. Head Neck. (1994) 16:165–72. doi: 10.1002/hed.2880160211 8021137

[6] Mazzaferri EL. Papillary thyroid carcinoma: factors influencing prognosis and current therapy. Semin Oncol. (1987) 14:315–32. 3306936

[7] Ortiz S Rodríguez JM Soria T Pérez-Flores D Piñero A Moreno J. Extrathyroid spread in papillary carcinoma of the thyroid: clinicopathological and prognostic study. Otolaryngol–Head Neck Surg Off J Am Acad Otolaryngol-Head Neck Surg. (2001) 124:261–5. doi: 10.1067/mhn.2001.113141 11240987

[8] Andersen PE Kinsella J Loree TR Shaha AR Shah JP. Differentiated carcinoma of the thyroid with extrathyroidal extension. Am J Surg. (1995) 170:467–70. doi: 10.1016/S0002-9610(99)80331-6 7485734

[9] Chung SR Baek JH Choi YJ Sung TY Song DE Kim TY. Sonographic assessment of the extent of extrathyroidal extension in thyroid cancer. Korean J Radiol. (2020) 21:1187–95. doi: 10.3348/kjr.2019.0983 PMC7458864 32729261

[10] Mahajan A Suryavanshi S Shukla S Vaish R Agarwal U Cruz AD. Active Surveillance of Low-Risk Papillary Microcarcinoma of the Thyroid in Indian Scenario: Are we Ready for it? A Narrative Review. Indian J Endocrinol Metab. (2022) 26:119–26. doi: 10.4103/ijem.ijem_501_21 PMC9302424 35873936

[11] Mahajan A Agarwal U Padashetty S Shukla S Smriti V Rastogi S. A narrative review of the role of cross-sectional imaging in the management of thyroid carcinoma: Imaging guidelines and T-CIRADS. Cancer Res Stat Treat. (2022) 5:490. doi: 10.4103/crst.crst_300_21

[12] Mahajan A Vaish R Sable N Arya S Kane S Cruz AD. 391P Incremental value of preoperative CT in the surgical management of papillary thyroid cancer. Ann Oncol. (2016) 27:ix121. doi: 10.1016/S0923-7534(21)00549-4

[13] Mahajan A Shukla S Ankathi SK Shukla A Vaish R Suryavanshi S. Imaging recommendations for diagnosis, staging, and management of cancer of the thyroid, parathyroid, and salivary glands. Indian J Med Paediatr Oncol. (2023) 44:159–74. doi: 10.1055/s-0042-1760403

[14] Chakrabarty N Mahajan A Basu S D’Cruz AK. Comprehensive review of the imaging recommendations for diagnosis, staging, and management of thyroid carcinoma. J Clin Med. (2024) 13:2904. doi: 10.3390/jcm13102904 38792444 PMC11122658

[15] Price DL Shah JP. Surgery for locally extensive carcinomas of the thyroid gland. Oper Tech Otolaryngol-Head Neck Surg. (2009) 20:7–17. doi: 10.1016/j.otot.2009.02.008

[16] Djalilian M Beahrs OH Devine KD Weiland LH DeSanto LW. Intraluminal involvement of the larynx and trachea by thyroid cancer. Am J Surg. (1974) 128:500–4. doi: 10.1016/0002-9610(74)90263-3 4417694

[17] Cody HS Shah JP. Locally invasive, well-differentiated thyroid cancer. 22 years’ experience at Memorial Sloan-Kettering Cancer Center. Am J Surg. (1981) 142:480–3. doi: 10.1016/0002-9610(81)90379-2 7283051

[18] Kim H Jung HJ Lee SY Kwon TK Kim KH Sung MW. Prognostic factors of locally invasive well-differentiated thyroid carcinoma involving the trachea. Eur Arch Otorhinolaryngol. (2016) 273:1919–26. doi: 10.1007/s00405-015-3724-4 26198285

[19] Hoang JK Branstetter BF Gafton AR Lee WK Glastonbury CM. Imaging of thyroid carcinoma with CT and MRI: approaches to common scenarios. Cancer Imaging. (2013) 13:128–39. doi: 10.1102/1470-7330.2013.0013 PMC3613791 23545125

[20] Kwak JY Kim EK Youk JH Kim MJ Son EJ Choi SH. Extrathyroid extension of well-differentiated papillary thyroid microcarcinoma on US. Thyroid Off J Am Thyroid Assoc. (2008) 18:609–14. doi: 10.1089/thy.2007.0345 18578609

[21] Hu S Zhang H Zhong Y Agyekum EA Sun Z Ge Y. Assessing diagnostic value of combining ultrasound and MRI in extrathyroidal extension of papillary thyroid carcinoma. Cancer Manag Res. (2022) 14:1285–92. doi: 10.2147/CMAR.S350032 PMC8976480 35378782

[22] Wang JC Takashima S Takayama F Kawakami S Saito A Matsushita T. Tracheal invasion by thyroid carcinoma: prediction using MR imaging. AJR Am J Roentgenol. (2001) 177:929–36. doi: 10.2214/ajr.177.4.1770929 11566708

[23] Seo YL Yoon DY Lim KJ Cha JH Yun EJ Choi CS. Locally advanced thyroid cancer: can CT help in prediction of extrathyroidal invasion to adjacent structures? AJR Am J Roentgenol. (2010) 195:W240–244. 10.2214/AJR.09.3965 20729422

[24] Lee DY Kwon TK Sung MW Kim KH Hah JH. Prediction of extrathyroidal extension using ultrasonography and computed tomography. Int J Endocrinol. (2014) 2014:351058. doi: 10.1155/2014/351058 25525431 PMC4265702

